# A Rare Case of Xanthogranulomatous Pyelonephritis with Spontaneous Renocolic Fistula and IVC Thrombosis

**DOI:** 10.1155/2021/3604017

**Published:** 2021-09-07

**Authors:** Daniele Sforza, Leandro Siragusa, Matteo Ciancio Manuelli, Linda De Luca, Bruno Sensi, Simona Grande, Renato Argirò, Marco Nezzo, Massimo Villa, Michele Grande

**Affiliations:** ^1^Department of Emergency, Policlinico Tor Vergata Hospital, Rome, Italy; ^2^Department of Surgery, Tor Vergata University of Rome, Rome, Italy; ^3^Department of Diagnostic Imaging and Interventional Radiology, Tor Vergata University of Rome, Rome, Italy

## Abstract

Xanthogranulomatous pyelonephritis (XGPN) is a rare disorder affecting the kidney which can fistulise to the colon in exceptional cases. We herein report a case of XGPN with renocolic fistula and large vessel thrombosis presenting with sepsis and pulmonary embolism. Preoperative diagnosis and strategic planning resulted in successful management. A 64-year-old woman presented to the emergency department with abdominal pain and a septic condition, corroborated by venous thromboembolism. Workup diagnosed a left renal abscess with calicocolic fistula. Scintigraphy confirmed a nonfunctioning left kidney. The patient underwent inferior vena cava filter placement and staged surgery. The first, damage control procedure was a loop ileostomy. Ten days later, when the patient's conditions improved, she underwent left nephrectomy and left colectomy with primary anastomosis. Finally, a year later, the ileostomy was closed. At follow-up, the patient was well, with unremarkable renal function. Scrupulous diagnostics, multidisciplinary decision making, and staged intervention have been key to optimal outcome.

## 1. Introduction

Xanthogranulamatous pyelonephritis (XGPN) is a rare cause of chronic renal disease, characterized by suppuration and accumulation of lipid-laden macrophages in the renal parenchyma [1]. A really uncommon sequela is the renocolic fistula, which is usually a consequence of primary bowel pathologies [2, 3].

We present a rare case of XGPN complicated by spontaneous renocolic fistula. Our patient presented with flank pain and clinical signs of deep vein thrombosis (DVT) and sepsis.

## 2. Case Report

A 64-year-old female was admitted to our emergency department for abdominal pain, fever, and signs of left lower limb DVT. She had no past medical or family history of thrombophilic disorders.

On physical examination, there were tenderness in the left lumbar region with mass and pain, edema, and swelling of superficial veins as well as increased skin temperature of the left lower limb. Her body temperature was 38.2°C, heart rate 105 beats/min, and blood pressure 100/60 mm Hg. Blood tests revealed anemia, leukocytosis, and increased C-reactive protein and D-dimer.

**Figure 1:**
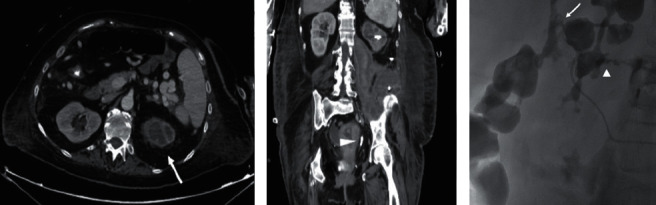
(a) Postcontrast CT images showed hypoperfusion of the left kidney with thinning of the cortex; dilatation of the left kidney excretory system with a “bear's paw sign” is also depicted suggesting diagnosis of xanthogranulomatous pyelonephritis (arrow); (b) postcontrast coronal reconstruction demonstrated ureteral stone (arrowhead) in the distal tract of the left ureter without significant dilatation of the proximal tract; (c) oblique projection pyelography after puncture of the inferior calix of the left kidney demonstrated leakage of contrast media with a double fistulous path form the pelvis directed anteriorly to the left colon (arrow) and posteriorly to the collection in the retroperitoneal collection (arrowhead).

Ultrasonography confirmed the diagnosis of DVT; a CT scan revealed pleural effusion, massive pulmonary embolism, inferior vena cava thrombosis, voluminous retroperitoneal abscess of the left psoas muscle and left kidney with irregular borders, hypodense areas, and a staghorn calculus suspicious for XGPN (Figures 1(a) and 1(b)).

The patient received two units of packed red blood cells and fluid resuscitation therapy; she was also started on empiric piperacillin/tazobactam antimicrobial therapy and enoxaparin sodium. After a multidisciplinary team meeting in the emergency area, the patient underwent an interventional radiologic procedure consisting of caval filter placement, left nephrostomy, and percutaneous drainage of the perinephric abscess; during the radiological procedure, contrast injected via the percutaneous drainage revealed a communication between the abscess, the lower pole calyx, and the descending colon (Figure 1(c)).

Therefore, the clinical and radiological features were suggestive of chronic pyelonephritis with spontaneous renocolic fistula, voluminous perinephric abscess, and reactive large vein thrombosis. We decided to treat the patient with a temporary ileostomy procedure, delaying definitive major surgical treatment, in order to ameliorate the clinical conditions. The postoperative course was uneventful, with a progressive improvement of general status. The early broad-spectrum antibiotic therapy was successful, with progressive decrease of inflammatory markers. Renal scintigraphy demonstrated a nonfunctioning left kidney, and a CT scan revealed resolution of pulmonary embolism. After 10 days from the first procedure, left nephrectomy and segmental left colectomy with terminoterminal anastomosis were performed. The patient had a good postoperative recovery and was discharged on day 38 from admission to the emergency department. On follow-up, the patient was asymptomatic and urinalysis, renal function tests, and colonoscopy were normal. After 12 months, the patient underwent ileostomy closure without complications.

**Figure 2:**
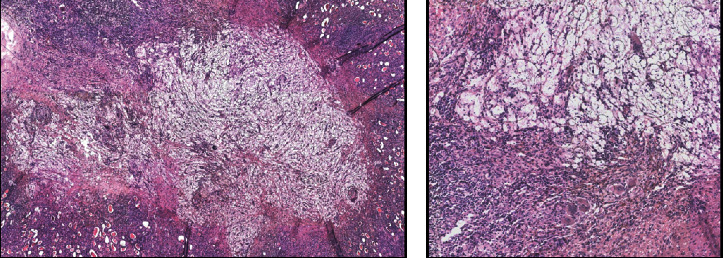
(a) H & E 4x: acute pyelonephritis, with abscessualization of the calices and pelvis and xanthogranulomatous chronic inflammation; (b) H & E 10x: detailed image of the typical multinucleated giant cells in the context of XGPN.

Histopathological examination of the kidney showed an intense and widespread acute pyelonephritis, with abscessualization of the calices and pelvis and xanthogranulomatous chronic inflammation, with multinucleated giant cells, lipid-laden macrophages, and acute inflammatory cells, as well as loose “xanthoma” cells (Figure 2). Yellow tissue was present around many calices. There was evidence of papillary microadenoma but not carcinoma. These features were diagnostic of XGPN. The resected colon was the site of acute focally abscessed, diffuse serositis.

## 3. Discussion

XGPN was first described by Schlagenhaufer in 1916 [4]. This process is histopathologically characterized by suppuration, renal parenchyma destruction, and the presence of lipid-laden foamy macrophages [5]. Macroscopically, the affected kidney appears as a mass of yellow tissue with focal necrosis and hemorrhage. Histiocytes destroy the tissues, which leads to chronic pyelonephritis [6]. XGPN is more common in females and can occur at any age, although it is more usual during the fifth and sixth decades [7]. It affects both the kidneys with equal frequency, and the incidence is estimated to be approximately 8% in all kidneys removed or biopsied for inflammatory diseases (excluding glomerulonephritis) [8].

Precise etiology is unclear; however, it is thought to occur in association with infection and/or chronic urinary tract obstruction such as staghorn calculi [8]. Urinary tract calculi are present in 70–79% of patients with XGPN [9]. Other contributory factors include diabetes mellitus, immunocompromised states, and abnormal lipid metabolism. The disease can behave and appear somewhat like a renal cell carcinoma.

In this condition, inflammation of the renal parenchyma, with increased pressure inside the kidney, results in necrosis of the cortex. The consequent abscess formation is the cause of damage to neighboring organs; local invasion of adjacent structures has also been described, with cases of spleen, pancreas, or duodenum involvement or development of renocolic, renocutaneous, and renobronchial fistulas [10].

Classification of XPGN recognizes three stages based on disease extension:

- Stage I: nephric, disease is confined to the renal parenchyma only
- Stage II: nephric and perinephric, the disease process involves the renal parenchyma along with perinephric fat
- Stage III: nephric and perinephric, disease extending into the adjacent structure or diffused in the retroperitoneum

Classical features are those of an acute suppurative disease, frequently associated with a perinephric abscess. Clinical presentation is typically similar to pyelonephritis with dull, persistent flank pain, and associated fever, dysuria, malaise, and weight loss. Pneumaturia can be a presenting symptom. Evolution may be insidious with progressive weight loss, malaise, persistent renal sepsis, dehydration, anemia, and uremia. Rarely, there is cutaneous fistulation. Laboratory tests are nonspecific, with anemia and increased inflammatory markers being a common finding. Urine cultures are often positive for *Escherichia coli* and *Proteus* species.

Although renocolic fistulas were described by Hippocrates in 460 B.C., with a renal abscess invading the intestinal tract, the first modern report of renocolic fistula was in 1841 by Rayer. Since then, the advent of antimicrobial drugs and CT scan has made the pathology infrequent [11]. Etiology can be spontaneous, iatrogenic, or, rarely, traumatic, such as following severe abdominal trauma. Spontaneous renocolic fistulae are a consequence of primary chronic kidney disease, such as tuberculosis or hydatid cyst or malignancy [12, 13].

Renocolic fistulae are an uncommon complication of XGPN with no large case series in the literature to the best of our knowledge [13–15]. Anatomically, the left colon's posterior wall, devoid of serosa, is directly opposite the anterior surface of the left kidney. The inflammatory process can cause perforation of a thinned cortex and subsequent drainage of infected urine and necrotic material from perinephric abscess into the colon, resulting in a renocolic fistula [13, 14]. In XGPN, the kidney can be strongly adherent to adjacent structures, such as the psoas muscle and adrenal gland, with numerous new blood vessels extending from the inflamed kidney. Suspicion of a sinus or fistula should be assumed for purposes of surgical treatment when the renal parenchyma is adherent to structures such as the large or small bowel or the diaphragm [8, 13].

Radiological diagnosis of renocolic fistula in the setting of XGPN is often difficult in the absence of clinical suspicion, but some radiological signs are suggestive; frequently, the CT scan demonstrates the presence of a pelvic staghorn calculus and round areas of low density surrounded by a rim of contrast medium in the renal parenchyma (bear paw sign) [16, 17]. Radiological investigations allow diagnosis through CT scan, antegrade pyelography, barium enema, or, if there is cutaneous extension, fistulogram [1].

Parson et al. reported the first series of patients with fistulation in XGPN; since then, there have been few reports of this rare complication [8]. One case of preoperative diagnosis with retrograde pyelography was described. In another series, Majeed et al. similarly describe patients with renocolic fistulae as a complication of XGPN [18]. In this report, the renocolic fistulas were identified incidentally during surgery while the preoperative imaging did not detect radiological signs of fistulation.

In the majority of cases, the renal parenchyma is completely destroyed and nephrectomy is the definitive treatment. The affected colon is resected, and when conditions permit, a primary anastomosis is performed [6]. However, if the involved bowel was macroscopically normal and the fistulous opening was very small, primary suture closure is also possible. Usually, this is a unilateral process; however, in the rare case of bilateral XGPN, successful bilateral partial nephrectomy has been performed [19]. In our case, the extensive renal damage encouraged us to proceed with nephrectomy. Our choice to perform a defunctioning temporary ileostomy to exclude and protect the renocolic fistula and delay nephrectomy and colonic resection was conditioned by the patient's very poor general status; the patient was admitted with sepsis, severe anemia, and pulmonary embolism and also needed placement of an inferior vena cava filter because of extensive thrombosis.

The designation of XGPN as a separate entity is important because it is not only often recognizable as a clinicopathological syndrome but also unique among the inflammatory conditions of the kidney in closely mimicking the clinical, radiological, and even histological features of renal cell carcinoma [13].

Prognosis largely depends on the underlying etiology, renal failure stage, and the patient's general status.

XGPN is an unusual type of chronic pyelonephritis, resulting from chronic obstruction of the urinary tract by an infected stone. Spontaneous renocolic fistulas are extremely rare in this setting, especially after the progress of antimicrobial therapy and renal stone treatment. Scrupolous imaging is necessary for accurate diagnosis. The singularity of our case lies in the contemporaneity of pyelonephritis, renocolic fistula, pulmonary embolis and large vein thrombosis in the context of sepsis. The complexity of the clinical condition has imposed a successful two-stage management of the surgical complications of XGPN. [20]

## Data Availability

Data supporting reported results can be found in the database of Policlinico Tor Vergata (http://www.ptvonline.it). Data are protected and access availability must be obtained.
